# Unlimited Paid Time Off Policies: Unlocking the Best and Unleashing the Beast

**DOI:** 10.3389/fpsyg.2022.812187

**Published:** 2022-03-24

**Authors:** Jessica de Bloom, Christine J. Syrek, Jana Kühnel, Tim Vahle-Hinz

**Affiliations:** ^1^Faculty of Social Sciences, Tampere University, Tampere, Finland; ^2^Department of HRM&OB, Faculty of Economics and Business, University of Groningen, Groningen, Netherlands; ^3^Department of Business Psychology, University of Applied Sciences Bonn-Rhein-Sieg, Rheinbach, Germany; ^4^Department of Occupational, Economic and Social Psychology, University of Vienna, Vienna, Austria; ^5^Department of Organizational, Business, and Social Psychology, Psychologische Hochschule Berlin, Berlin, Germany

**Keywords:** self-determination theory, freedom, flexibility, organizational policy, autonomy, social exchange theory, holiday, leave

## Abstract

Unlimited paid time off policies are currently fashionable and widely discussed by HR professionals around the globe. While on the one hand, paid time off is considered a key benefit by employees and unlimited paid time off policies (UPTO) are seen as a major perk which may help in recruiting and retaining talented employees, on the other hand, early adopters reported that employees took less time off than previously, presumably leading to higher burnout rates. In this conceptual review, we discuss the theoretical and empirical evidence regarding the potential effects of UPTO on leave utilization, well-being and performance outcomes. We start out by defining UPTO and placing it in a historical and international perspective. Next, we discuss the key role of leave utilization in translating UPTO into concrete actions. The core of our article constitutes the description of the effects of UPTO and the two pathways through which these effects are assumed to unfold: autonomy need satisfaction and detrimental social processes. We moreover discuss the boundary conditions which facilitate or inhibit the successful utilization of UPTO on individual, team, and organizational level. In reviewing the literature from different fields and integrating existing theories, we arrive at a conceptual model and five propositions, which can guide future research on UPTO. We conclude with a discussion of the theoretical and societal implications of UPTO.

## Introduction

Recent headlines in major newspapers and online media illustrate that unlimited paid time off policies are currently fashionable and widely discussed by HR professionals around the globe (Reeves, 2021): “Unlimited holiday: The rise of leave without limits,” “Unlimited vacation policy: Why employers should consider it,” “The ugly truth about unlimited holidays,” “Why unlimited vacation days is a scam,” “Unlimited vacation sounds amazing. It can burn workers in the end” and “Four lessons about unlimited vacation.” These examples also showcase the paradoxical effects which have been described in popular media. On the one hand, paid time off is considered a key benefit by employees (AICPA, 2018) and unlimited paid time off policies (UPTO) are seen as a major perk which may help in recruiting and retaining talented employees. On the other hand, some early adopters reported that employees took less time off than previously, presumably leading to higher burnout rates. Accordingly, HR professionals proposed measures and boundary conditions which may ensure that UPTO unfolds its assumed benefits while preventing any harmful side-effects. However, theoretical reasoning and empirical evidence is missing to show and explain why these measures work. Therefore, we set out to build a theoretical model on UPTO and its underlying processes and formulated propositions to explain if and under which conditions UPTO can benefit or harm individual employees, the team, and the company.

**FIGURE 1:**
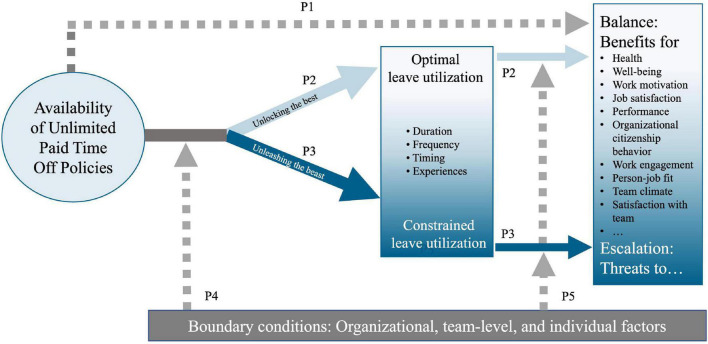
Conceptual model on effects of Unlimited Paid Time Off Policies (UPTO).

The COVID-19 pandemic and steep rise in remote work sparked even more interest in UPTO and related flexible work arrangements with potentially wide-ranging implications for performance management (e.g., Results Only Work Environments). In this conceptual review, we will synthesize the available theorizing and very scarce empirical evidence to predict the effects of UPTO on employee health, well-being, motivation, and job performance. We developed a conceptual model (Figure 1) that depicts how the effects of UPTO should exert their influence on employees from the theoretical lens of self-determination theory (Ryan and Deci, 2000) and social exchange theory (Homans, 1958; Blau, 1964). Specifically, we propose that UPTO can “unlock the best” and engender feelings of autonomy which in turn lead to favorable outcomes for employees and ultimately the organization. At the same time, UPTO utilization is shaped by negative social processes which may “unleash the beast” and result in harmful outcomes for employees and the organization. Finally, we propose boundary conditions of UPTO which facilitate “unlocking the best” in employees and conditions that may rather “unleash the beast” and harm individual workers, the team, and the organization.

Our manuscript is divided into five sections. Firstly, we define and place UPTO in a historical and international context. Secondly, we focus on leave utilization as a key construct which translates UPTO from a hypothetical option into concrete action (i.e., taking leave). Thirdly, we describe the two parallel processes which we termed “unlocking the best” and “unleashing the beast” which are set in motion simultaneously by making UPTO available. Fourthly, we delineate boundary conditions on individual, team, and organizational level which can affect whether and how UPTO will be utilized and thus the degree to which UPTO has beneficial, neutral or even harmful consequences. Fifthly, we discuss the theoretical and societal implications of UPTO.

### Work Intensification, Flexibilization, and Leisure Scarcity - A Need for Unlimited Paid Time Off Policies?

How did UPTO become such a hot topic within the field of Human Resource Management? How did managers around the world came to think that providing workers with an unlimited amount of paid leave might be a good idea? Below, we describe the historical and societal developments which gave rise to UPTO.

A globalized 24/7 economy, automation, digitalization, and technological advancements such as smart mobile Information and Communication Technology devices which enable employees to work anywhere and at any time have led to structural changes in the way work is organized, carried out, and experienced (Green, 2004; Kubicek and Korunka, 2017). In today’s “Industry 4.0” (Schwab, 2017), most employees work in the service industry or conduct knowledge work, requiring them to engage in emotional labor, lifelong learning and efficient task and time management (Jarvis, 2007; Grandey and Melloy, 2017). For many workers, a primary work task is “non-routine” problem solving and job performance is determined by the employee’s ability to acquire, share, and utilize knowledge (Reinhardt et al., 2011). The very concept of work has become flexible, accompanied by a change in the nature of employment relationships such as a proliferation of temporary, project- and platform-based work, and high levels of job insecurity (e.g., Burchel et al., 2002; Rofcanin and Anand, 2020). Spatial and temporal boundaries between work and non-work domains increasingly vanish, even more so after the COVID-19 pandemic hit and people work from home for significant shares of their working time, while work pace and workload increase, leading to the perception of accelerated working lives (Rosa, 2015; Piasna, 2018).

Research on work trends in recent decades has shown that Dumazedier’s (1967) vision of a “leisure society,” characterized by abundant opportunities for relaxation, distraction from work, and personal development, did not materialize hitherto for most workers. In fact, his predicted decreases in individual annual working time have only occurred in certain industries such as manufacturing, whereas increases have occurred in other sectors such as the service industry (for an insightful historical overview of working time developments over the past centuries see Wilensky, 1963). Job demands have even intensified, and people perceive their working days nowadays as intense (Boxall and Macky, 2014; Kubicek and Tement, 2016): Time pressure is high and increasing, and many people feel pushed to work faster and longer to meet deadlines (Baethge et al., 2019). Concerning leisure time, we nowadays witness polarization along demographic and socio-occupational lines. For instance, time-use data from Canada and the Netherlands shows that workload for paid and unpaid workers has risen over the last two decades, while the amount of free time has declined (Zuzanek et al., 1998). Along similar lines, results from the latest European Working Conditions Survey show that 22% of workers report that they work during their free time several times a month to meet work demands (Parent-Thirion et al., 2017) and for many workers, life is characterized by feelings of “time famine” or “time squeeze” (Schor, 1991). Glorieux et al. (2010) identified a significant share of the workforce as the “harried leisure class,” highly educated high-income workers who constantly feel short in time. Accordingly, leisure is increasingly seen as a scarce commodity that needs to be spent in efficient ways, resulting in phenomena such as “time deepening” (Godbey, 1976).

Furthermore, most modern work happens behind a computer and output became less tangible than before. After the decline of factories and the production of goods in the western world, the need to coordinate the work efforts of large groups of workers via physical presence at the same time and place has steadily decreased. Today, many workers produce services and knowledge, making it more difficult for employers to exert control and closely monitor employees’ work tasks and output. Consequently, the responsibility for the strict regulation of work tasks and working times has - at least partly - shifted back from employers to employees.

These structural changes in working life and the changing nature of work together with the perceived scarcity of leisure time have led to a heightened need and desire to manage one’s work and free time autonomously and preferably to have more leisure. This trend is reflected in (re)newed interest in alternative ways of working and increased opportunities to take time off from work when needed and desired.

As early as in the 1970s, many companies already implemented (more or less) drastic changes toward flexible working times and/or shorter weekly working hours. Yet research on these new types of working has remained scarce, rendering inconclusive evidence (for a summary of early studies see Bird, 2010). Rather than an actual absence of beneficial effects, the ambiguity in these findings may be due to the great variety of working time arrangements under investigation as well as differences in the uptake of these arrangements in practice (i.e., availability versus utilization). Conceptual frameworks that can guide empirical investigations are urgently needed to investigate the effects of different flexible working time arrangements in depth.

In this manuscript, we focus on UPTO as a concrete, specific, and very timely example of a new working time arrangement. UPTO is not only relevant because it fits the Zeitgeist of modern working life, but also because it is universally applicable to all workers. This also distinguishes UPTO from other types of flexible work arrangements (i.e., flexible working hours, compressed work weeks, reduced work hours and/or flexibility in work location) which workers are allowed to but do not need to use (for a review, see Shifrin and Michel, 2021). Whereas many work-non-work policies are geared at specific workers or life phases workers undergo, when UPTO is introduced, it substitutes all previous, classical leave policies and is thus automatically applicable to all workers.

### The Rise of Unlimited Paid Time Off Policies

In the next sections, we will define UPTO, provide a few concrete examples of UPTO which have been introduced in practice and then move on to briefly describe the historical background of leave legislation as well as an international comparison on leave policies. We deem this overview key to understand the importance of the societal, organizational, and individual context which can affect the utilization of leave.

We define UPTO as unlimited and sporadic paid time off from work during which an employee can be away from work and is not required to conduct any work-related tasks with negotiable boundary conditions such as timing, length, and requirements regarding coordination and performance. The term “sporadic” is important here, because it distinguishes UPTO from structural adjustments of weekly working hours. Thus, people’s contractually defined weekly working hours remain unchanged, just like the location for working, but employees are provided with the opportunity to take time off from work whenever desired while receiving their full wage. In principle, as indicated by the label of being unlimited, there is no maximum number of days off that can be taken. In practice, many companies still communicate a maximum length of single leave episodes or state that leave can only be taken if approved by the team, supervisor, and/or if workload allows. Therefore, UPTO has often been criticized in the media for not being truly unlimited or even being a scam.

As previously mentioned, UPTO has been widely discussed as a flexible working time arrangement among practitioners in human resource management. In September 2020, an internet search, for instance, yielded more than 88,700 hits for the search term “unlimited paid time off” and 36,700 hits for “open paid time off.” Many human resource managers came to view UPTO as an attractive tool in attracting and managing modern knowledge workers. In several industries, the whole concept of fixed working hours is frowned upon and seen as a relic from times when performance could easily be measured in “minutes on task.” Particularly in the tech industry, HR managers claim that creative and cognitively demanding work requires new ways of working and of measuring a person’s input and output (e.g., Jordan et al., 2022). Therefore, UPTO and abandoning a fixed number of annual leave days resembles the *Zeitgeist* of modern work without temporal or spatial boundaries. In addition, a shortage of workers in the tech industry also forms a strong incentive to create attractive workplaces for employees. In the “war of talent,” UPTO has been portrayed as a means to attract and retain talented employees. Consequently, numerous tech companies such as Netflix, Hubspot, Dropbox, or Kronos have introduced UPTO. As UPTO is not limited to specific types of jobs, companies in other industries have followed soon. In the following section, we provide a brief historical and international overview of paid and unpaid leave which helps us to conceptualize and contextualize UPTO.

### Conceptualizing and Contextualizing Unlimited Paid Time Off Policies

Work and leisure are often portrayed as opposites. In fact, work, defined as purposeful activities requiring mental and/or physical exertion and carried out in the public domain in exchange for wages (Wilson, 1996:23), can be seen as a precondition for the existence of leisure. That is, work and leisure are interdependent. But sometimes work and leisure are even difficult to distinguish, and research finds that people without work do not perceive leisure as such (Wilson, 1996; Ciulla, 2000). Accordingly, leisure scientists have long struggled to define leisure, frequently resulting in somewhat arbitrary or circular definitions of leisure as “time outside work” or simply as the opposite of work (for a discussion of this challenge, see Beatty and Torbert, 2003). Historically, leisure as a concept emerged when the physical space of work moved outside people’s homes. With rising levels of wealth, leisure was initially only a privilege of the upper classes. With labor movements and unionization of workers during industrialization came greater protection of workers’ rights, including a reduction in weekly working hours. The right to leisure which guarantees free time to everyone was established by the Universal Declaration of Human Rights signed by 48 member states of the United Nations in 1948. In modern times, boundaries between work and leisure have again become blurred and life domains have merged. Due to modern technology, most people carry their work “in their pocket” around the clock, and work-related emails are the first thing people have a look at when opening their eyes in the morning. Work and leisure are no longer seen as antithetical but flow into and complement each other in a dynamic relationship (Beatty and Torbert, 2003). Since the start of the COVID-19 pandemic, work and private life have become even more intermingled: A great share of the working population works from home and structural and physical boundaries between life domains have vanished completely. Thus, legal definitions and legislation on leisure time and rest periods have been implemented to delineate work and leisure and protect worker’s health and well-being.

Across the world, work is regulated by laws which also regulate the right to and timing of rest periods. Paid time off is defined as a pre-defined number of days each year that an employee is allowed to be away from work while still receiving full wage. Legislation in the United States and the EU exemplifies well the extreme differences which exist regarding annual paid time off across the globe. Most industrialized states in the world can be placed somewhere between these two extremes. In the United States, workers do not have the legal right to paid annual leave, treating leave as a perk rather than a worker right (Ray and Schmitt, 2007). Each state in the United States has own labor laws, and in most states, employers can decide whether they grant paid leave to their employees or not. Consequently, 26% of Americans have no access to paid leave (U.S. Bureau of Labor Statistics, 2018) and the United States’ average is only eight vacation days per year. Moreover, the right to leave is also unequally distributed among United States workers favoring high wage, highly educated full-time workers (Ray et al., 2013).

In the European Union, legal rights to at least 4 weeks of paid vacation per year were established in 2003 (DIRECTIVE 2003/88/EC, 2003). EU countries must comply with this directive and EU companies can only grant more, but never less than 4 weeks of leave. Compared to the United States, with no legal statutory right to leave, European companies have a generous leave policy in place (i.e., annual leave plus several special types of leave for life events such as moving, sickness, or death of a family member). Still, many Europeans save up leave days for personal emergencies, leading to unused vacation days at the end of the year (which can only be saved for a limited time), and suboptimal use of the leave granted by the employer. This problem is likely even more prominent in countries where employees do not have the legal right on sick leave, but a fixed amount of leave which is to be used for vacations, sick leave, and personal emergencies. Introducing UPTO might be seen as a solution to this problem, because people no longer feel the need to save days for special circumstances, leading to fewer accrued leave days at the end of the year (which also constitutes a liability for companies), and better recovered employees. In addition, what should be kept in mind is that while the implementation of UPTO is anchored on an organizational level, the individual availability of UPTO can be perceived very differently by employees. Therefore, we would like to direct attention toward employees’ individually perceived availability and accessibility of UPTO when the impact of UPTO is assessed. That is, we suggest that part of how UPTO become effective is driven by the degree to which employees perceive UPTO to be available to them, whether and how they make use of the policy.

## Unlimited Paid Time Off Policies and Leave Utilization

In our conceptual model on UPTO, leave utilization is key in translating UPTO into concrete actions (see Figure 1) - first and foremost regularly taking time off when desired and/or needed. Research on working life policies has shown that accessibility of policies is not the same as their utilization (Ford and Locke, 2002; Kirby and Krone, 2002). While the availability of UPTO can indeed have beneficial effects on workers’ well-being by providing employees with some “peace of mind” (i.e., the idea that the policy would be available to them in times of need; P1 in Figure 1), UPTO should moreover affect well-being, health, and performance once workers actually make use of the policy and utilize the policy optimally. To illustrate this argument with an example: Some human resource managers have described in the media that workers actually took fewer holidays after the introduction of UPTO. It is possible that workers in these companies nevertheless report higher job satisfaction than before they had UPTO, because they feel that they could take time off whenever they like. However, if workers feel happier but do not actually take leave, UPTO cannot lead to profound or lasting benefits. Therefore, it is also essential to focus not only on the outcomes, but also carefully monitor and understand the underlying processes which transform the policy into potential benefits for health, well-being, and performance. Below, we present a short description of how leave utilization might differ between persons, and how these differences may relate to differences in employees’ health, well-being, motivation, and performance.

Leave utilization can vary regarding the duration, frequency, and timing of leave periods as well as regarding recovery experiences during leave. Regarding duration and frequency of leave, evidence from research on vacations suggests that longer leaves do not necessarily have stronger or longer lasting effects on health and well-being (De Bloom et al., 2008). For instance, both long weekends (4 days) and 5- or 10-day domestic holidays can significantly improve well-being (Kühnel and Sonnentag, 2011; De Bloom et al., 2012) and even the beneficial effects of 6-month sabbaticals fade soon after returning to work (Davidson et al., 2010). So, the frequency of leaves seems to be somewhat more important than the duration of single leave episodes. Still, both are key indicators of leave utilization. Accordingly, we suggest using both the duration and frequency of taking leave before the introduction of UPTO as a benchmark, as these indicators provide important information on the impact of UPTO. For example, if the total number of leave days taken decreases, this may be an indication of employees experiencing barriers to taking leave such as pressure to finish work tasks colleagues are depending on (Barber et al., 2019). If under UPTO the same number of free days is taken and greater variance between people emerges in terms of frequency, this could mean that people have increasingly adapted leave to their personal needs.

Some implications regarding leave timing can be drawn from research on breaks at work. For example, research on energy management strategies suggests that breaks are particularly useful in times of low energy and increased distress to prevent further resource depletion (Fritz et al., 2011; Zacher et al., 2014). Thus, especially in times of low energy resources, such as after a busy period at work or after an important deadline, taking leave may be beneficial (e.g., Sonnentag, 2018).

Research on stress and recovery after work has provided evidence on specific aspects in terms of experiences during leave that are beneficial for recovery. Four recovery experiences have been shown to have beneficial effects for employees in terms of well-being: detachment (mentally distancing oneself from work), relaxation (low activation and increased positive affect), control (ability to choose between different activity options), and mastery (challenging experiences and the opportunity to learn new things) (Sonnentag and Fritz, 2007, 2015). In the DRAMMA model, which combines evidence from psychology and leisure sciences (Newman et al., 2014), this list was extended by two additional experiences: meaning (activities that provide a sense of purpose) and affiliation (activities that foster the feeling of relatedness to others). The empirical evidence suggests that leave which fosters these experiences is positively related to optimal functioning, i.e., higher vitality, life satisfaction, subjective health, and lower depressive complaints, need for recovery, tension, and strain (Sonnentag et al., 2017; Kujanpää et al., 2020; Virtanen et al., 2020). We propose that UPTO enables employees to take leave more regularly, spontaneously, and for longer time periods, which should stimulate beneficial recovery experiences. On the basis of the limited research to date, we propose that UPTO implemented so that duration, frequency, and timing of leave periods can be adjusted to individual needs should relate to positive outcomes for employees.

## Unlocking the Best and Unleashing the Beast

In the following sections, we will provide a theoretically and empirically guided overview of the effects of UPTO on employees’ health, well-being, work motivation, and performance. Figure 1 summarizes our conceptual model, and shows that we aim to describe the effects of UPTO as a function of releasing the beneficial potential of autonomy and setting in motion social processes, which we refer to as “unlocking the best” and “unleashing the beast,” grounded in self-determination theory (Ryan and Deci, 2000) and social exchange theory (Homans, 1958; Blau, 1964), respectively. Our model aligns with and can explain “paradoxes” that regard autonomy (Mazmanian et al., 2013) and flexible work arrangements (Cañibano, 2019), showing that well-intended policies can also result in (unintended) negative outcomes for employee and employer.

Our model illustrates that with the introduction of UPTO, two processes are likely to be evoked simultaneously. The first process, which we call *unlocking the best*, describes the most likely intended beneficial effects of UPTO: Employees are given autonomy over their leave, which should lead to beneficial outcomes. The second process, which we call *unleashing the beast*, illustrates the paradoxical situation in which well-intended policies may turn into unwanted outcomes. Employees are granted autonomy over their leave, but detrimental social processes are activated (such as normative pressure within a work group, informal expectations about taking leave), which corrupt the idea of autonomy and turn the freedom of taking leave into an obligation of not taking (too much) leave.

The first process, unlocking the best, is grounded in self-determination theory (Ryan and Deci, 2000), which states that autonomy is a key ingredient for a fulfilled life. Satisfaction of people’s innate need for autonomy leads to higher work engagement, better health, well-being, work motivation, and performance. The second process - unleashing the beast - is based on social exchange theory (Homans, 1958; Blau, 1964), which highlights that under UPTO, taking leave constitutes a process that is heavily shaped by social interactions. These social processes can curtail the intended individual freedom into social obligations associated with an atmosphere of guilt, excessive responsibility for organizational or team goals and consequently harmful effects for employees, such as poorer health, well-being, work motivation, and performance. It is important to note that we propose that both processes are at play simultaneously, and that individual-level, team-level, and organizational factors will determine which process will prevail (see boundary conditions described below).

We propose that the processes we term “unlocking the best” and “unleashing the beast” are partly mediated by *leave utilization*. Regarding the assumed positive pathway, availability of UPTO may lead to beneficial outcomes directly (P1) and by enabling workers to adjust their leave utilization to their personal needs which in turn liberates psychological resources and positive emotions, resulting in greater well-being and energetic resources (P2). On the other hand, detrimental social processes may restrict leave utilization, and, for instance, inhibit optimal timing of leave by putting teams’ work goals before individuals’ recovery goals. This can drain people’s energetic resources and in the long-term lead to feelings of exhaustion (P3). We will describe these processes in greater detail below.

In the outcome part of our conceptual model on the right, we describe potential outcomes of availability of UPTO. Positive effects resulting from UPTO are reflected in a balance between individual and organizational needs such as higher job satisfaction, well-being, and work engagement, a better work-non-work balance, as well as more organizational citizenship behaviors. Negative effects resulting from UPTO are reflected in an imbalance between individual and organizational needs, which is likely to result in short-term higher work engagement and job satisfaction but also in long working hours, more working during leisure time, rumination about work after office hours, and higher work-non-work conflicts. In the long term, negative effects may prevail as temporary strain reactions cannot be reversed and people must perform while still feeling tired (Meijman and Mulder, 1998). This process further drains emotional and cognitive resources and depletes personal energy, ultimately leading to serious threats to well-being, health problems such as burnout, sleeping problems, anxiety, or depression. In the next section, we describe both processes of our conceptual model in detail and present preliminary findings from research supporting our propositions.

### Unlocking the Best

Autonomy regarding leave is seen as a key element of healthy work and attractive jobs. In representative surveys across industrialized nations, shorter working hours and extended amounts of free time are increasingly seen as desirable. When asked whether employees would prefer higher salaries or more vacation days, the majority of workers vote for more leisure (e.g., AICPA, 2018; Ver.di, 2019), mirroring the shift in priorities from consumption of physical goods toward services and experiences (Pine and Gilmore, 1998). Therefore, UPTO enabling employees to take agency over their work time, is also often communicated as an asset to attract and retain talented employees (Hill et al., 2008).

Job autonomy, defined as “the degree to which the job provides substantial freedom, independence, and discretion to the employee in scheduling the work and in determining the procedures to be used in carrying it out” (Hackman and Oldham, 1976: 162), is considered the core mechanism which can bring about the positive effects of UPTO. Job autonomy has been shown to be an essential ingredient for work-related well-being and performance. It helps employees to achieve goals at work, and can facilitate personal growth (e.g., Hackman and Oldham, 1976; Spector, 1986). Indeed, major theories in the field of work psychology have something to say about autonomy, and also outside the work context, autonomy is seen as a basic human need and its satisfaction as a key mechanism helping people to thrive and flourish in life (Deci and Ryan, 2008). Thus, the relationship between job autonomy and well-being of employees is explained by the satisfaction of autonomy as a basic human need.

In abandoning the use of a fixed amount of leave, companies aim to establish a culture of psychological ownership. Just like self-employed entrepreneurs, employees are considered capable of managing their work tasks and striking an optimal balance between the needs of the company and their personal needs. UPTO may signal trust of the company in employees, may empower them, reaffirm their status and sense of self as accomplished professionals trusted to make responsible use of UPTO. This could benefit both the employees and the company. Studies have indeed shown that providing employees with higher levels of autonomy makes them feel accountable and more committed to their work (e.g., Spector, 1986). This in turn positively affects organizational outcomes such as greater financial returns, customer satisfaction, productivity, lower employee turnover, and fewer accidents (Harter et al., 2002). In conclusion, we expect that UPTO leads to benefits for employees (e.g., greater well-being, work engagement), because it fosters satisfaction of autonomy as a basic human need, as proposed in self-determination theory (Deci and Ryan, 2008). This leads to our first proposition:

• P1: Availability of UPTO provides the satisfaction of autonomy as a basic human need and therefore can directly lead to benefits for employees such as greater well-being and work engagement.

Furthermore, we assume that the relationship between availability of UPTO and beneficial outcomes is also partly mediated by leave utilization. Control over the timing and duration of leave enables employees to align their work better with their personal needs and experience a better balance between work and non-work life. According to recovery research, job control regarding the timing of recovery episodes is important for optimal well-being (Sonnentag et al., 2017). As the job demands people experience vary across time, depending on the job tasks they must perform, their need for recovery also varies (Sonnentag and Zijlstra, 2006). Moreover, individual characteristics (e.g., stress resilience, personality traits such as neuroticism, hardiness, and resilience) may determine employees’ optimal workload and need for recovery (Sonnentag et al., 2010; Kraaijeveld et al., 2014). Employers introducing UPTO assume that employees can recognize if and when they need to recover and act accordingly by taking time off. UPTO could foster the optimal timing and duration of taking leave based on personal preferences, work characteristics, and person characteristics. For example, during the current COVID-19 pandemic UPTO could help employees by allowing them to take leave to adjust to burdens associated with the pandemic (e.g., childcare, homeschooling). Enabling people to take time off from work whenever needed may provide them with a means to optimize their personally preferred patterns of effort and recovery.

Leave from work, as a prolonged episode of recovery from work and mental disengagement from work, enables employees’ psychobiological systems to return to baseline levels and reestablish full working capacities and well-being (Meijman and Mulder, 1998; Sonnentag and Fritz, 2015). Numerous empirical studies in occupational health psychology have indeed shown that recovered workers are healthier, more committed to their work, and perform better (e.g., De Bloom et al., 2008; Binnewies et al., 2010; Kühnel et al., 2017). Autonomy in taking leave according to one’s personal needs might also foster the leave experiences of psychological detachment and control. For example, adjusting the start of a vacation to an unexpected pressing deadline reduces the number of unfinished tasks when finally start their vacation. Leaving behind a “clean desk” is beneficial in terms of mentally distancing oneself from work (Syrek et al., 2017), and reduces work-related rumination. Additionally, autonomy to take leave when desired heightens control over free time and vacation activities. For instance, UPTO may help employees to take leave when the weather is nice or an important event takes place and supports engagement in personally meaningful hobbies (e.g., sailing or running a Marathon). Thus, UPTO offers higher control in the choice of activities during leave days and thereby improves the quality of leave experiences. This leads to our second proposition:

• P2: Beneficial outcomes of UPTO are partly explained by optimal utilization of leave. Specifically, we propose that availability of UPTO enables employees to adjust their leave to their personal needs, resulting in optimal duration, timing, and frequency of leave days as well as better leave experiences. This in turn leads to beneficial well-being and performance outcomes.

### Unleashing the Beast

Contrary to the proposed direct link between availability of UPTO and beneficial outcomes, we do not propose a direct link between availability of UPTO and negative outcomes. That is, we consider it unlikely that the mere availability of UPTO can deteriorate employee well-being or performance. Even though there is some research showing that work-family policies can be perceived unfair by people who do not have children and therefore do not make use of certain policies (called the “family-friendly backlash;” Parker and Allen, 2001), UPTO is not restricted, specifically tailored to or particularly relevant to certain groups of workers. Instead, we assume that potential negative effects of UPTO unfold via suboptimal leave utilization. We explain this process via social exchange theory (Homans, 1958; Blau, 1964).

Following social exchange theory (Homans, 1958; Blau, 1964), UPTO can be seen as an inducement of the company which requires a contribution to the company from the employees’ side, i.e., UPTO may create a social obligation toward the employer. Taking advantage of UPTO may thus lead to a feeling of obligation or even guilt toward the employer. In return for UPTO and the freedom it supposedly provides, the organization can expect the employee to be an ideal worker (Putnam et al., 2014). Following Kelliher and Anderson’s (2010) and Cañibano’s (2019) argumentation, we suggest that UPTO becomes a part of a psychological contract between the employees and the employer, and entails certain tacit expectations regarding the appropriate leave behavior under UPTO. People’s vision of an ideal worker is thereby shaped by their professional and workplace norms (Wieland, 2010), and empirical evidence suggests that this often means working overtime and making sacrifices for the employer. For instance, research on flexible work arrangements and technology which enables workers to work more flexibly, has shown that people often tend to put in more hours, experience more conflicts between work and private life (Peters et al., 2009), and perceive work as intensified (Kelliher and Anderson, 2010; Mazmanian et al., 2013). Individuals may internalize organizational goals, which promotes overcommitment or self-endangering behaviors (Peters, 2001; Deci et al., 2016) and that limit employees’ leisure time at the expense of the company. According to Deci et al. (2016) these behaviors include prolonging working hours, intensifying working hours, using substances for recuperation, taking stimulants, working despite illness, lowering the quality of work, and failing to observe safety standards. These behaviors tend to occur due to high work demands and interestingly are particularly common in workplaces with high levels of autonomy (Baeriswyl et al., 2014). Self-endangering work behaviors also go hand in hand with health problems seriously impaired recovery from work-related stress (Deci et al., 2016).

In the absence of formal rules on leave and without a specification of an exact number of leave days per year, workers may be inclined to exercise greater levels of control over others, leading to “concertive control” (Barker, 1993; Ter Hoeven et al., 2017). Concertive control is characterized by strong identification with the team and/or the organization, strict informal rules and norms within teams, and punishment and reward exercised by the team. For instance, research has shown that self-managing teams which are granted more autonomy increase their control over individual team members (Barley and Kunda, 1992; Ezzamel and Willmott, 1998; Sewell, 1998). An endless number of vacation days means that employees have no guidance on how much leave is appropriate. When no formal rules exist, employees will look for informal rules communicated by their supervisor or team members. Normative pressures within teams can induce an employee to conform to the team’s values and courses of action. Consequently, employees are likely to imitate their peers’ behaviors, because these behaviors signal the norms deemed appropriate (Gino et al., 2009). Descriptive norms (what is actually happening at the workplace) have a stronger effect on behavior than injunctive norms (what ought to be happening at the workplace), even when the descriptive norm does not align with the injunctive norm (Kallgren et al., 2000). In popular media, this process has been described and companies with a “No vacation policy” have been criticized for disregarding this effect. Under UPTO, employees can take as much time off as they wish (injunctive norm), but the number of days that will ultimately be taken depends on what other team members do (descriptive norm). Depending on the company culture, the team culture and the personality of the supervisor, wide differences between people and teams may emerge in terms of the utilization of UPTO. This could contribute to a work environment where the utilization of UPTO is discouraged (see also McDonald et al., 2005).

In a similar line of argumentation, it could be argued that leave is owned by an individual worker under standard (non-unlimited) leave policies. In most companies, additional leave hours can be bought, and excess leave hours can be exchanged for extra salary. Workers leaving an organization usually need to be paid out all unused leave hours. But under UPTO, leave becomes a shared good. If one worker takes more leave, this may imply less leave for another. Under UPTO, a worker can no longer compensate the company and/or their team for additional leave taken by giving up some salary. This means that they are at the mercy of their colleagues and supervisors for granting them additional free time at the expense of the group. Under UPTO, leave changes from an individual trading good into a collective good.

Finally, we would like to zoom in on the paradoxical role of autonomy. In her essay on what she calls the “performance society,” Lynn Berger (2020) refers to the downside of autonomy as the “perversion of freedom.” Referring to philosopher Han’s (2015) essay on the “burnout society,” Berger states that the shift from external control of work through an employer to the employee leads to an ever-increasing need for self-optimization. External prohibition, command, and regulation at work are increasingly replaced by internal initiative, motivation, and self-discipline. As this discipline comes from the inside rather than from an external force, resistance is impossible, resulting in self-exploitation. This exploitation of the self is more efficient than exploitation by an external force because it is actually perceived as freedom. The exploiting and exploited become one.

Regarding leave utilization these detrimental social processes imply that fewer vacation days may be taken than under policies with a fixed number of vacation days per year. Several companies which had introduced UPTO canceled the policies because they could indeed see this happening (e.g., Gateley, 2018; Sweeney, 2019). This phenomenon bears a strong resemblance to what has been described as “leavism.” Leavism refers to employees’ tendency to take leave when they are actually sick or unable to complete their work in time (Hesketh and Cooper, 2014). Under UPTO, some employees may take leave, but actually just work from home to save commuting time, run errands or take care of family obligations during the working day. A change toward mainly shorter leaves and a decrease of longer leaves would be indicative of people not feeling free to make optimal use of UPTO.

Interestingly, evidence from breaks at work shows that employees have difficulties in recognizing their need for recovery and tend not to take breaks if they have the autonomy over taking breaks (Henning et al., 1989). Sonnentag (2018) coined the term “recovery paradox” to describe the empirical finding that recovery processes are particularly impaired when they are needed most, that is, when employees face high job stressors. Under UPTO, employees may similarly fail to initiate leave days when their self-regulatory resources diminish, and initial signs of distress occur. This process could be further amplified by organizational structures that couple high autonomy with high work demands and responsibility to meet these demands.

Teams may also struggle to jointly decide who is granted what amount of leave. Team members are often dependent on each other’s work and a day off for one team member can mean additional work for another. Negotiating an optimal balance between team members can be tough and carry the potential for conflict. Even when teams may jointly arrive at an agreement on how many days off each employee is granted, some employees may need more days due to personal circumstances or desires (e.g., family emergencies, need for recovery, traveling the world). It may be challenging to argue for this within a team while keeping a fair distribution of leave among team members, i.e., the same amount of leave for all. To be granted permission to get more days off than their team members, employees may feel forced to disclose information about their personal circumstances requiring them to take time off. Leave turns from a right into a dispensation from the company, controlled by the team and/or supervisor. This means, compared to fixed leave policies, under UPTO, employees may be controlled more by workplace norms, and their own view of the ideal worker which affects leave utilization and the content of leave experiences. Being granted leave by the team may be associated with feelings of guilt for leaving the colleagues to deal with stressful situations or feeling obliged to make up for taking leave when back at the workplace. Thus, UPTO can lead to ruminating or worrying about work during the vacation and reduce mental detachment. Additionally, leave may only be taken if the moment is right for the team, which reduces the options regarding leave activities (which may depend on the time of the year, and therefore reduces control over leave activities). This leads to our third proposition:

• P3: Negative outcomes under UPTO are partly explained by constrained utilization of leave. Specifically, we suggest that detrimental social processes hinder employees from adjusting leave to their personal needs resulting in not recognizing or ignoring the need to take a leave and suboptimal leave duration, frequency, timing, and leave experiences.

## Boundary Conditions that May Shape the Effects of Unlimited Paid Time Off Policies

Below, we provide an overview of boundary conditions which support or hinder optimal utilization of UPTO and boundary conditions which affect the pathway from leave utilization to outcomes. We emphasize that these factors merely serve as examples in the broad spectrum of potentially relevant boundary conditions.

### Individual Level

We will discuss three individual factors that affect whether and how leave is utilized and translated into beneficial outcomes. Firstly, studies show that women generally utilize flexible work policies and vacation leave more intensely than men (Maume, 2006). According to Maume (2006), this difference is partly explained by traditional expressions of work-family priorities in which men take fewer leaves because they are more concerned about job security and coordination issues at work whereas women are more concerned about their families. Rather than a true gender effect, the differences found may thus be explained by work centrality. Work centrality refers to the importance a person assigns to working in comparison to other life domains such as leisure, family or religion (Paullay et al., 1994). People who view work as central to their identity are likely to utilize leave to a smaller extent (i.e., shorter and less frequent leaves) than people who have a more balanced identity, including other life domains and roles as well.

Secondly, we propose that people with a high need for segmentation between life domains make relatively little use of flexibility in time or place compared to people with a low need for segmentation (Shockley and Allen, 2010). We propose that people with a high (vs. low) need for segmentation may use UPTO to the same extent, but for different purposes and thus with different consequences. People with a strong segmentation preference may more likely use UPTO as a recovery opportunity, because they have strong boundaries between work and non-work domains. These well-established boundaries ensure that when they take leave, they will not engage in work-related activities during their non-work time. People with a low need for segmentation, however, are at greater risk of using UPTO to engage in what we have introduced as “leavism” above. They will more likely continue to work during their leave, and they may even take leave in times of high workload just to tend to their work from home and schedule their time more efficiently (e.g., by saving travel time).

Thirdly, personality traits such as neuroticism or openness may influence both an employee’s need for recovery from job stress and their desire to travel to discover new places and meet new people, respectively, and thus whether available UPTO will be utilized. Recovery-related self-efficacy may determine whether workers benefit from taking time off. Recovery-related self-efficacy refers to “an individual’s expectation of being able to benefit from recovery time and recovery opportunities” (Sonnentag and Kruel, 2006: 202). It is an important predictor of recovery from job stress (e.g., Park and Lee, 2015; Park and Kim, 2019). Accordingly, we propose that people who lack recovery-related self-efficacy will less likely and less extensively make use of UPTO. In addition, employees with higher recovery-related self-efficacy may benefit more from taking leave than people with lower recovery-related self-efficacy.

### Team Level

Group level processes play an important role in UPTO utilization. Decisions on leave are often shifted from supervisors to the team level. This means, depending on the workload, personal preferences, and considerations of fairness, teams might decide jointly who can take time off from work, when, and for how long. Some teams may establish rules in which the whole team needs to approve the plans of each team member whereas other teams shift this responsibility to their team leader. Factors such as team maturity (i.e., how long does a team work together), diversity and location (i.e., remote or on-site) may either simplify or complicate the process of establishing norms within a team and having constructive discussions on how to organize leave-taking within a team.

While UPTO might at first sight seem more suitable for knowledge workers, there are several companies around the world that have introduced UPTO even though their workers’ performance and output depends on physical presence. For instance, to cure patients or ensure satisfied call center clients, a team needs to collaborate to achieve their joint goal(s). Occupation rate (i.e., services are provided to clients/patients around the clock) is key in this endeavor. This means that employees need to negotiate the timing and duration of their leave with their colleagues. This is actually true for any kind of leave policy. Consequently, an employee can only take off if another employee covers their shift. As workers are more dependent on each other to achieve their work goals and perform well, the social exchange process is key.

Overall, such processes can potentially result in conflicts within the team and between the team and the supervisor. Technical tools and expertise that would help teams to reliably predict workload and occupation rates required to handle the workload on specific days or times of the year might be beneficial in order to prevent team conflicts under UPTO.

An important factor in determining whether individual employees can take leave relates to structural interdependence. According to Courtright et al. (2015), interdependence includes both task and outcome interdependence, meaning that team members depend on one another for access to critical resources and coordinated action in order to establish well-functioning workflows. Moreover, performance expectations, goals, feedback, and rewards are often on the team level. Consequently, individual team members may feel high levels of responsibility in making sure that their team achieves common goals and completes projects in time. This may restrict the freedom of individual team members to take leave and withdraw temporarily from investing in the team’s shared goals. The stronger the interdependence of a team, the lower utilization of leave is expected to be.

A related construct with similar effects is team identity. Team identity is defined as a bond (personal, cognitive, emotional, and behavioral) between an individual and their team (Henry et al., 1999) and represents the extent to which an individual perceives oneness with their team (Ashforth and Mael, 1989). Research has shown that people with a strong team identity are inclined to follow and invoke team norms (Somech et al., 2009). It is likely that people who identify strongly with their team and the team’s shared goals will be less inclined to utilize UPTO for their personal benefit as this may harm the team’s goals of achieving certain work tasks within a specific time frame. The opposite effect may occur in teams with a strong “recovery culture” (Sonnentag et al., 2021), i.e., teams with a shared awareness that recovery is important and valuable. These teams may encourage and enable each other to take leave whenever needed.

### Organizational Level

Organizational factors also affect how employees utilize UPTO. We will describe three important factors: organizational culture, leadership, and workload. Firstly, based on Cameron and Quinn’s (2006) framework, a clan culture defined as an environment promoting caring for the individual worker and positive relationships, can be expected to foster optimal leave utilization compared to a market culture, which stimulates within-group competition rather than cooperation. Empirical support of this argument has been reported by Timms et al. (2015) who have demonstrated that a non-supportive organizational culture (i.e., expectations that employees work long hours and that careers will be negatively impacted if employees make use of flexible work arrangements) is related to non-use of flexible work arrangements. Similarly, Peetz and Allan (2005) found that flextime can lead to a long hour working culture.

Secondly, leadership plays a crucial role in discouraging or encouraging utilization of UPTO, either by directly communicating expectations or by acting as role models. We expect that both empowering and transformational leadership which provide subordinates with individualized consideration and intellectual stimulation support subordinates in terms of leave utilization, whereas transactional leadership focusing on compliance by subordinates through both rewards and punishments may create an environment that restrict that subordinates’ use UPTO according to their personal needs. Research has shown that a market culture and transactional leadership are associated with “obsessive passion,” defined as a rigid persistence in work activities and an uncontrollable urge to work hard, resulting in long working hours and conflicts in other life domains (Vallerand, 2010). This closely resembles what we have described as self-endangering work behaviors and overcommitment.

Research has also shown that workers tend to recognize their supervisors’ orientation toward health (Franke et al., 2014) and, for example, imitate their supervisors’ behaviors in terms of segmentation between life domains (Koch and Binnewies, 2015). Communication by supervisors about policies shapes what Ter Hoeven et al. (2017) refer to as “acquired rules.” These rules are defined as beliefs which guide employees’ decisions regarding the use or non-use of work–life policies. That means, supervisors serve as important role models which will shape the utilization of UPTO in their subordinates.

Thirdly, high workload, urgency and frequent, tight deadlines are very decisive as to if and how UPTOs are used. Research has also shown that individuals with longer tenure in the organization, supervisory responsibilities, and with coworkers who utilize flexible work are more likely to utilize flexible work policies than are workers without tenure, supervisory responsibilities, and who do not perceive their workgroup as using the newly acquired flexibility (Lambert et al., 2008). This suggests that workers need to feel secure and perceive the organizational culture as being supportive of flexible work policies in order to actually make use of such policies. Transparency about rules within the company can guide employees in establishing the right amount of leave for them.

Fourthly, another important element on the organizational level is a system to monitor leave. While it may seem tempting for companies to abandon all rules and simplify leave registration, a registry is essential to monitor and intervene, particularly if employees take too little leave. In the European context, it is also important to note that the law requires a minimum number of leave days to be taken every year. Therefore, companies have the legal duty to record leave and prove that they adhere to the legal guidelines. Moreover, such a registration system can also help teams to coordinate their work tasks and occupation rates.

This argumentation for individual, team-level, and organizational factors above leads to the following propositions:

• P4: Individual, team-level, and organizational factors affect whether and how UPTO is utilized. For example, on individual-level, employees with high work centrality may take fewer leave days than employees with a lower work centrality. On team level, teams with a supportive recovery culture may stimulate their team members to take more regular/longer leave. On organizational level, software systems which help workers to predict workload and required occupation rates on the work floor can help workers to coordinate their leave periods and take time off when needed and possible.
• P5: Individual, team-level, and organizational factors affect the relationship between leave utilization and outcomes. For example, on individual level, employees with a high recovery-related self-efficacy may benefit more from taking leave than employees with lower recovery-related self-efficacy. On team level, team members with high interdependency may shame colleagues for taking leave during a busy period at work, thereby offsetting the beneficial effects of leave taking. On organizational level, high workload, and tight deadlines right after holiday periods may prevent beneficial vacation effects to translate into lasting well-being and performance benefits.

## Discussion

In this conceptual review, we focus on the newly emerging HR policy of UPTO. Building on and extending earlier work on the paradox of autonomy (Mazmanian et al., 2013) and flexible work (Cañibano, 2019) and integrating self-determination and social exchange theory, we have developed a conceptual model and five propositions on the effects of UPTO leading to benefits or threats for worker’s well-being, health, and performance. We propose the effect of availability of UPTO unfolds via two simultaneously occurring processes which either release the benefits of autonomy resulting in higher well-being, motivation, and performance, or trigger detrimental social process which limit leave utilization and with negative long-term consequences for individuals, teams, and the organization. Central in our model is the utilization of UPTO which translates sheer availability of UPTO into consequences. Finally, we propose that several boundary conditions at individual, team, and organizational level are at play that either foster or inhibit the optimal utilization of leave in terms of leave frequency, duration, timing, and experiences, and that shape whether workers benefit from utilization of leave. In developing our model, we draw on earlier research findings on autonomy and flexible work scattered across various disciplines and integrate them in a coherent framework with the help of two major theories: self-determination theory (Ryan and Deci, 2000) and social exchange theory (Homans, 1958; Blau, 1964). Below, we elaborate on the theoretical implications of our work and reflect on the wider societal implications of UPTO.

### Theoretical Implications: Potential Extension of Motivation Theories

Self-determination theory forms one of the two core elements of our model, suggesting that UPTO enables employees to satisfy their need for autonomy which in turn should directly lead to optimal functioning. However, self-determination as a theory focuses on the individual worker and cannot fully explain the “unleashing the beast” part of our model, which relates to social exchange theories. More importantly, we believe that self-determination theory does not sufficiently capture situations in which people are both intrinsically motivated (i.e., voluntarily participate in work activities out of enjoyment), *and* work due to external pressures such as described in the “unleashing the beast” part of our model. In line with Berger’s reasoning on the “perversion of freedom,” internalized pressures for self-optimization and self-exploitation, and the “emergent theory of neoclassical calling” (Bunderson and Thompson, 2009), we speculate that self-determination theory may be further developed to include what we would call “escalated motivation.” Escalated motivation could account for the short-term adaptive (e.g., “walking the extra mile” for the company, high work engagement) and long-term non-adaptive outcomes of autonomy (e.g., long working hours, burnout) and may represent a unique combination of intrinsic motivation and introjected regulation. We think that such an adaptation would fit the context of modern working life in which external control by the organization is often replaced by internal control. Building on Michel Foucault’s analyses of neoliberalism, Casalini (2019) describes basically the same phenomenon of self-exploitation when stating “The neoliberal individual is invited to think of himself or herself as free, but in fact is dependent on the imperatives of the neoliberal social environment” (p. 136). In combination with the increasing pressure to enjoy work and experience one’s job as meaningful (Berkelaar and Buzzanell, 2014; Graeber, 2018), this new type of “escalated motivation” may also explain rising levels of burnout (Aumayr-Pintar et al., 2018; Gallup, 2019). Thus, a combined consideration of the benefits of autonomy and possible corruptions of these effects due to negative interfering social processes, might provide a theoretical lens that explains paradoxical effects of flexible work arrangements in modern working environments.

Regarding the detrimental social process of UPTO, we have mainly focused on situations in which employees are too committed to the organization and invest (too) much effort in their work. But in fact, the opposite can happen, too. That is, if an employee feels exploited by the organization, they may reduce their input to the organization. As an example, an employee may make more use of UPTO after having learned that they did not get the expected promotion and associated pay rise. This may re-establish a perceived effort-reward imbalance, but obviously has direct harmful consequences for the organization (Siegrist, 2002; Van Vegchel et al., 2005). It is also interesting to note that this abuse of UPTO is an often-raised fear in the media.^1^ And who would keep working anyways if you do not need to, but could be on holidays year-round? This question taps into our ideas on human nature and the nature of work. And there is an answer derived from studies on the universal basic income and research on hypothetical and actual lottery winners. These studies show that around two-thirds of people would keep working even if they would not need a salary to make a living (for an overview and summary of these studies, see Hüffmeier and Zacher, 2021).

Relatedly, we have not considered positive social exchange processes in our model. However, it is also possible that availability of UPTO leads team members to feel trust and gratitude toward each other, which in turn may lead to beneficial team-level outcomes such as a positive team climate. Accordingly, social processes may also complement the “unlocking the best” process occurring at the individual level.

### Societal Implications: Unlimited Paid Time Off as a Modern Form of Piecework?

Unlimited paid time off in its most liberal form means complete freedom to take time off whenever desired. Employees could drastically reduce their weekly working hours or decide to work only a couple of weeks per month or a few months per year. As long as their work gets done, employers should in principle accept this utilization of UPTO. When UPTO is taken to this extreme (which in practice rarely happens), it implies maximum flexibilization of working times. In fact, it would mean totally abandoning fixed working times. There has been already heightened interest in flexible working arrangements regarding *when* and *where* work is done (e.g., Putnam et al., 2014; Rofcanin and Anand, 2020). UPTO implies that employees could even decide *if* they work at all. Consequently, employers would need to implement management practices to ensure that the job gets done at the point in time they want it done. Most of the companies that have implemented UPTO thus far do indeed have management systems in place which are clearly based on output. That is, these companies often have HR practices such as management by objectives with clearly formulated organizational goals and systems in which supervisor and employee jointly set measurable objectives, progress toward these objectives is closely monitored, and attainment of the objectives within a pre-set time frame is evaluated and rewarded.

We assert that working under UPTO in this extreme (and hypothetical form) may be comparable to piecework in which employees get paid a fixed piece rate for an action performed or product completed, irrespective of the time they worked on it. In modern work, the “piece” would be attaining a pre-defined objective such as a project completed, a product delivered, or a deal signed with a new client. If working hours and physical presence at the workplace no longer serve as a criterion for productivity, employment contracts may drastically change or may become obsolete. Consequently, work arrangements may increasingly become non-standard “gigs” (Gandini, 2018). Under UPTO, employees may increasingly become or made into entrepreneurs or freelancers.

### Unlimited Paid Time Off Policies as a Process Evolving and Changing Over Time

Last but not least, we would like to mention that the introduction of new HR policies is a dynamic process unfolding across various levels within the organization. For instance, when investigating UPTO, it would be important to compare employees with and without UPTO (between-person comparison) and employees before and after UPTO introduction (within-person comparison). Between persons, higher variance in leave duration and frequency in the group with UPTO compared to a group with regular leave schemes would be indicative of higher autonomy under UPTO and the path of “unlocking the best” in our model. Comparing the time before and after UPTO introduction in the same persons, stability in the number of leave days taken and increasing variance in leave frequency would suggest that UPTO helps employees to adjust their leave to individual needs and preferences.

Following Van Mierlo et al. (2018), the introduction of a new HRM policy like UPTO can be seen as a process that evolves and changes over time. Newly introduced HRM practices change the behavior of various actors at the workplace. This in turn affects how these practices play out and affect these different actors. Subsequently, this may lead policymakers and HRM managers to adapt the rules or introduce new policies and the process starts over again. This means that UPTO might be best represented and investigated within a multilevel framework. For instance, it is likely that individual-level relationships (e.g., positive relationship between autonomy and benefits of UPTO) are dependent both on team-level constructs (e.g., pressure to succumb to team norms) and individual-level constructs (e.g., being jealous of other team members’ leave taking). UPTO is also expected to evolve over time when the context changes and employees experiment with UPTO, experience how it influences their well-being, job performance, and private lives and adapt the way they utilize it. The ongoing pandemic and rise in telework may further speed up the process of companies introducing flexible work policies and potentially also UPTO.

## Conclusion

When work and free time become increasingly intertwined, leisure may become work and work may become leisure. For instance, leisure has been defined as an “experiential quality of one’s time when one engages voluntarily and intentionally in awareness-expanding inquiry” (Beatty and Torbert, 2003: 239). In business life, countless variations exist of the saying that you will not work a day in your life if you choose a job you love. Both views fall short of capturing the essence of the struggle modern workers undergo. While the first view seems to suggest that work as counterpart to leisure is characterized by activities which are neither voluntary nor enjoyable, the second disregards work which is undertaken to earn a living rather than for fun or to achieve a greater purpose in life. The upcoming years will show how employers and employees will negotiate, arrange and manage work and non-work life domains and how new trends in work arrangements such as UPTO and telework will affect the process of striking a balance between closely interconnected life domains.

In this conceptual review, we studied UPTO as an example of flexible work policies which can benefit or harm individual workers and the organization, depending on the boundary conditions which facilitate or hinder utilization of the freedom which these policies supposedly create. Whilst UPTO can increase employees’ feeling of control, accountability, and work engagement, it could also lead to self-endangering work behaviors, long working hours, and exhaustion. We therefore sketch two competing processes and boundary conditions. One process that builds on self-determination theory captures “balance” (“unlocking the best”) in which UPTO allows employees to shape their work-non-work balance because of the autonomy that such policy gives them, and the positive benefits associated therewith. The second process that is grounded in social exchange theory reflects “escalation” (“unleashing the beast”), because UPTO may spark detrimental social processes which constrain leave utilization and arouse feelings of uncertainty and guilt concerning the required completion of work. In addition, absence of formal rules may lead to newly emerging informal rules which are not communicated and increase social conflicts. We think that empirical research is indispensable to reveal how UPTO can be implemented so as to benefit both employers and employees. We hope that our propositions can guide research on this important emerging policy.

## Author Contributions

JB, CS, JK, and TV-H were involved in conceptualizing the review, collecting and reviewing the available literature, developing the manuscript, contributed to the article, and approved the submitted version.

## Conflict of Interest

The authors declare that the research was conducted in the absence of any commercial or financial relationships that could be construed as a potential conflict of interest.

## Publisher’s Note

All claims expressed in this article are solely those of the authors and do not necessarily represent those of their affiliated organizations, or those of the publisher, the editors and the reviewers. Any product that may be evaluated in this article, or claim that may be made by its manufacturer, is not guaranteed or endorsed by the publisher.

## Footnotes

1
https://www.foxbusiness.com/small-business/what-to-do-when-your-employees-abuse-your-unlimited-vacation-policy
